# Woman’s Health Protective Association

**Published:** 1894-03

**Authors:** 


### Woman's Health Protective Association.

At the recent Sanitary Convention at Harrisburg, a paper was read by the Director of Public Safety of Philadelphia, Mr. A. M. Beitler, in which he paid a handsome tribute to the recent public work of an organization, the Woman's Health Protective Association of Philadelphia, for the great interest they take in the city's cleanliness and for the success they have had in forwarding complaints to the proper bureau for registry. The Director of Public Safety believes that the women of Philadelphia, through this quite inexpensive organization, will yearly make their work tell on the city's good order and for the observance of sanitary regulations already provided; that they will be potent aids in preserving the health of Philadelphia. This city, by its plan, and its favorable building developments, of a single house to one family, should have the smallest death rate of any in the world, for in no large city are the bulk of the people so comfortably and decently housed. It only needs the education of the individual householder to do his or her part in every way, by vigilance and by voting, by setting an example both in personal care of premises and in reporting at once all nuisances in the neighborhood; an example, also, on the part of each voter in choosing loyal and business men to conduct the city's business in councils; it only needs this to have, in a few years, a transformed city, with clean water to drink, all places of pollution shown up and cleaned out, and not only the smallest death rate in the world but the most comfortable city to live in. All of this the women may bring about if they will be ceaselessly vigilant and fearlessly outspoken. *Journal American Medical Association.*

